# Impact of long-term cyanotoxin exposure on cattle: Biochemical, histological, and oxidative stress assessment

**DOI:** 10.14202/vetworld.2025.189-201

**Published:** 2025-01-27

**Authors:** Mounira Bensalem, Amina Amrani, Hadjer Zaidi, Fateh Sedrati, Omar Laouar, Zhi Wang, Hichem Nasri

**Affiliations:** 1 Laboratory of Biodiversity and Ecosystems Pollution, University of Chadli Bendjedid, El Tarf, Algeria; 2 Department of Life and Nature Sciences, Sciences Faculty, University 20 Août 1955 Skikda, Algeria; 3 Department of Research Project Monitoring, Thematic Agency for Research in Health Sciences, Oran, Algeria; 4 Laboratory of Sciences and Technology of Water and Environment, Mohamed Cherif Messaadia University, Souk Ahras; 5 Central Pathology Laboratory, Mutaeb Hospital, Sakaka, Al Jouf, Kingdom of Saudi Arabia; 6 Key Laboratory for Environment and Disaster Monitoring and Evaluation of Hubei, Innovation Academy for Precision Measurement Science and Technology, Chinese Academy of Sciences, Wuhan, 430077, China

**Keywords:** cattle health, cyanobacterial blooms, environmental pollution, lake des oiseaux, microcystin, one health, oxidative stress

## Abstract

**Background and Aim:**

Cyanobacterial blooms, driven by anthropogenic and climatic changes, pose significant ecological and health threats. This study investigates the long-term effects of microcystins (MCs), potent cyanotoxins, on cattle at Lake des Oiseaux, a Ramsar-listed wetland in Algeria. Aligning with the “One Health” framework, the research evaluates the biochemical, histological, and oxidative stress impacts of MCs on livestock as environmental sentinels.

**Materials and Methods:**

A herd of 40 cattle (20 exposed and 20 non-exposed) was studied during the summer bloom period of 2019. Blood and liver samples were analyzed to assess biochemical markers (ALT, AST, ALP, GGT, etc.), histopathological changes, and oxidative stress parameters (GPx, CAT, SOD, LPO and GSH).

**Results:**

Exposed cattle exhibited significant elevations in liver enzymes and oxidative stress markers, indicating hepatic inflammation and redox imbalance. Histological analysis revealed macrovacuolar steatosis, fibrosis, and bile duct dilatation. Antioxidant enzyme activities (GPx, CAT and SOD) were reduced, with notable depletion of GSH levels and increased lipid peroxidation. These findings reflect the cumulative cytotoxic effects of MC exposure. Non-exposed cattle showed no such changes.

**Conclusion:**

Long-term MC exposure disrupts liver function and induces oxidative stress in cattle, implicating significant risks for both animal and human health. The bioaccumulation of cyanotoxins in livestock emphasizes the urgent need for preventive measures, including water monitoring, restricted livestock access to contaminated sites, and farmer education. These strategies are vital to mitigate risks under the “One Health” approach, ensuring sustainable livestock and public health.

## INTRODUCTION

Cyanobacterial toxins in water pose a threat to both humans and animals [1], with high morbidity and mortality rates worldwide [2, 3]. There are only a few studies on free-range cows and sheep raised in natural grasslands facing significant risks [4, 5]. According to Yunes [6], Amrani *et al*. [7], regardless of the route of contamination, the presence of cyanobacterial cells is greater than blooms in areas with open waters, leading to a “One Health” issue [8–11]. There are over 275 structural congeners in microcystins (MCs) [12–14]. The most commonly identified types of microcystin-LR receptor (MC-LR) and microcystin-RR (MC-RR) [15–17]. MC-RR and MC-LR have been the subjects of numerous toxicological studies [2, 18]. Living cells can release cytotoxins into water, remaining active even after cell death or burst. They can last for a long time after the bloom has passed. However, only approximately 5% of cyanobacteria species produce cytotoxins [19–22].

Once in the bloodstream, MC travels to the liver and is transported across hepatocyte cell membranes by organic anion-transporting polypeptides. MC-LR causes cytotoxicity in hepatocytes by inhibiting protein phosphatase 1 (PP1) and 2A, leading to hyperphosphorylation, cytoskeletal disruption, cell degradation, and apoptosis [23, 24]. The metabolic formation of reactive oxygen species induces oxidative stress, apoptosis, and DNA alterations [25, 26]. Acute exposure to high MC concentrations causes hepatic damage [27]. It is critical to emphasize the harmful effects of cynotoxin bioaccumulation and biotransform [7], in which tissue and plasmatic concentrations exceed the toxin rates in the environment due to absorption through all routes of exposure [28–30]. In 1998, the World Health Organization (WHO) proposed a provisional guidance value of 1 μg/L for MC-LR in drinking water, which has been modified by many countries, including Finland and Mexico [15, 31]. The Australian and New Zealand governments established guidance values (4.2 μg/L M.C.s for cattle and 3.9 μg/L M.C.s for sheep) [32, 33].

The State of California Environmental Protection Agency established a subchronic action level of 3 μg/L for MCs [34] based on Jackson’s study [35]. The highest non-lethal dose observed was 3.7 mg/kg bw (body weight) of MCs, whereas the maximum tolerated dose for acute exposure in domestic animals was calculated to be 0.037 mg/kg MCs bw. The water intake of beef cattle is 0.07 L/kg/day, with an acute action level of 200 μg/L MCs [34]. Climate change may explain the increased frequency of cyanobacterial blooms in many aquatic ecosystems [36–38]. The Lake des Oiseaux (Northeast Algeria) is a Ramsar site of international importance (1999) that supports diverse, rare, and unique flora and fauna. Since 2013, the lake has experienced intense blooms of the toxic genus *Microcystis* spp. during the summer and autumn seasons. The bloom is moderately toxic, with a concentration of 0.062 mg/g, and contains more than 21 MC variants [34]. The degradation of the lake is caused by a variety of factors, including drying, climate change, anthropogenic pollution (discharges of domestic and industrial wastewater and pesticide residues), fishing, uncontrolled hunting, and overgrazing [39].

The possibility of a high-risk increase in toxic blooms caused by acquired modifications in cyanobacterial combinations in nutrient-rich lakes appears to be associated with current global climate changes, which may be triggered by human agricultural activities and other factors [40–43]. Agriculture and cattle breeding are the most well-known human activities in the study area. Contaminated water from Lake des Oiseaux is used for irrigation and cattle drinking. The MC transfer in grazing animals is poorly understood. The primary goal of this research is to investigate the various effects of daily water consumption from Lake des Oiseaux on 40 cows and bulls in the study area, which will be accomplished through a battery of toxicological tests on blood and liver tissues. This study is the first report of cattle exposed to cyanobacterial toxins in the country. This should be the starting point for several global preventive studies that use animal exposure as a sentinel.

## MATERIALS AND METHODS

### Ethical approval

This study did not include any studies involving human participants. The animals sampled were raised for human consumption. Two orders (August 1, 1984, and July 15, 1996) issued recommendations regarding sampling and slaughter conditions. To obtain an effective date for the trial, animals were subjected to antemortem, slaughter, and postmortem practices that were standardized and regulated by international animal welfare guidelines (Terrestrial Animal Health Code 2018, section 7, Art 7.5.1) and Algeria’s national executive decree No. 95-363 of November 11, 1995. This was done and ethically approved by the head veterinarian of the slaughterhouses.

### Study period and location

**Figure 1:**
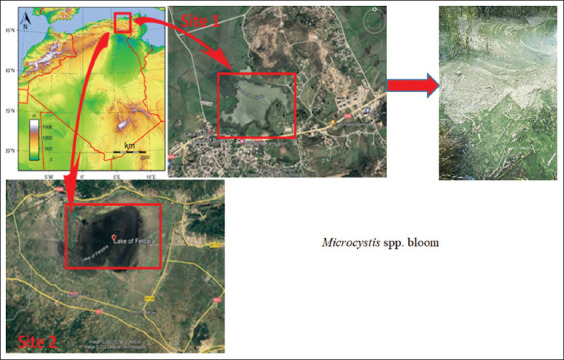
General location of the surveyed area and sampling sites: Site 1: Lac des Oiseaux, *Microcystis* spp. Bloom. Site 2: Lac de Fetzara (Google Earth, 2023).

This study was conducted from June 2019 to August 2019 at two sites, El Lake des Oiseaux and Lake de Fetzara. The shallow permanent freshwater “Lake des Oiseaux” or “Gaaraet Ettouyôur” (Figure 1), located in the northeast of Algeria (36°42’32” N 08°07’05” E), is considered a representative of the rare natural wetland type of the Mediterranean zone and has been classified as a Ramsar site (N°975) since 1999 and a part of El Kala National Park since 2003. Lake des Oiseaux has an oval surface that extends to the northwest by a pond tail with gently sloping banks. The lake covered 150 ha, had a maximum depth of 2.5 m, and deposited 1–3 cm of organic matter. According to Samraoui *et al*. [44], the lake’s environmental integrity is compromised by the different weights applied to it. The lake covers only 70 ha during the rainy season and 40 ha during the dry season, with a 20-cm organic matter deposit and a maximum salinity of 0.3‰ in September and October [44–46]. It is an important natural reserve that supports a large number of water bird species, particularly migratory ones, with over 38 species of aquatic avifauna recorded [47], as well as a diverse range of insects (Odonata) and vegetation (200 species of helophytes and hydrophytes) [48].

### Sample collection

The exposed group (EG) consisted of 30 local breed cattle (15 males and 15 females) raised on farms surrounding the lake, grazing and drinking in the area. Sampling was conducted during the summer of 2019, and 3 time points were combined (EG1 - June, EG2 - July, and EG3 - August). The duration of cyanobacterial blooming and the stability of water’s physicochemical factors, particularly temperature, affect the biochemical parameters. We defined a “Bloom” based on the visual criteria proposed by Diego [49] and detailed by Quiblier *et al*. [50] and Interstate Technology Regulatory Council [51]. At the same time, 10 other indigenous cattle were raised under the same conditions but in a non-polluted region called “Cheurfa” near Lake de Fetzara (Daira of Berrahal, Wilaya of Annaba) as a non-EG (NEG). The reference site, “Lake de Fetzara” (36° 43’ and 36° 50’ N, 7°24’ and 7°39’ E), which has been on the Ramsar list since 2002, involves the protection of this wetland of international interest with a remarkable diversity of fauna and flora, with one sampling time point in August of the same year. Each group consisted of five males (bulls) and five females (cows).

### Blood analysis

Blood samples (10 mL) were collected the day before slaughter to ensure animal welfare. Blood was drawn from the jugular vein into chemistry tubes. The serum was collected after centrifugation of the clotted blood and frozen at –20°C until the day of analysis. At the University Hospital Center CHU of Annaba, blood laboratory procedures were performed using standard biochemical techniques for evidence of hepatic inflammation and dysfunction and the lipid metabolism; alanine aminotransferase (ALT) (IU/L), aspartate-t-aminotransferase (AST) (IU/L), alkaline phosphatase (ALP) (IU/L), γ-glutamyltransferase (GGT) (IU/L), total protein (TP) (g/L), glucose (GLU) (mmol/L), albumin (ALB) (g/L), total cholesterol (TC) (mmol/L), triglycerides (TG) (mmol/L), total bilirubin (TBIL) (μmol/L), creatinine (CR) (μmol/L), and lactate dehydrogenase (LDH) (U/L) [52].

### Liver specimen collection

Liver tissue samples were obtained from the local slaughterhouses in the study area. Liver samples were divided into three groups: Histological examination, biochemical analysis, and antioxidant determination.

### Histopathological analysis

Multiple hepatic portions of fresh parenchyma were measured at 2 × 2 × 2 cm on average, carefully handled, and immersed in 10% neutral buffered aqueous formaldehyde for fixation. After automated dehydration, clearing, and embedding, 4–5 μm-thick sections were cut using a manual rotary microtome, dewaxed, and stained with hematoxylin and eosin (H&E). A routine light microscope was used for histopathological examination and assessment, and an HD camera was used to photograph the results [53]. Histochemical staining (Masson’s Trichrome) was used to assess inflammatory activity and fibrosis. H&E slides were classified for inflammation according to the METAVIR inflammation scoring system using the units presented in Table 1 [54].

**Table 1:** METAVIR inflammation and fibrosis scoring scale [54].

Grade scale	Interpretation of inflammation scoring	Interpretation of fibrosis scoring
0	No inflammation	No scarring
1	Minimal inflammation or occasional spotty necrosis	Fibrosis is confined to the portal tracts
2	Mild inflammation or minimal hepatocellular damage	Fibrosis extending outside the portal tract
3	Moderate inflammation with perceptible hepatocellular damage	Fibrosis extending outside the portal tract
4	Severe inflammation with noticeable diffuse hepatocellular damage	Bridging fibrosis: fibrosis extending and linking to central veins or other portal tracts

### Analysis of oxidative stress biomarkers

Previously, the liver fragments were identified, numbered, and placed in the freezer. They were thawed to begin the analysis. Using a buffer of 10 mM ice-cold Tris-HCl with 7.4 pH, a tissue aliquot was homogenized, vortexed, and centrifuged at 10,000 rpm for 10 min. The supernatants were removed for each analysis. The battery of oxidative stress biomarkers and TP content were measured spectrophotometrically, whereas the protein substance was measured using the Bradford dye-binding test with bovine serum albumin as a standard [55]. GSH content was quantified using Elman’s reagent (DTNB), a fluorescent reagent described by Weckberker and Cory [56]. The reaction mixture consisted of 500 μL of supernatant, 1.0 mL of Tris-ethylenediaminetetraacetic acid (EDTA) buffer (0.02 M, pH 9.6), and 25 μL of DTNB (0.01 M). The fluorescence at 412 nm was measured after complete mixing and incubation for 5 min at 25°C. The concentrations of GSH were calculated using a standard curve for pure GSH. GST activity was determined using 1-chloro-2,4-dinitrobenzene (CDNB) as a substrate following the methods reported by Habig *et al*. [57] and Zaidi *et al*. [58]. The reaction mixture includes 50 μL of supernatant, 1.05 mL of 100 mM Tris buffer (pH 7.4), 50 μL of 1 mM CDNB, and 50 μL of 1 mM GSH.

GST activity was determined after 2 min of observation of changes in absorbance at 340 nm. The enzymatic activity of glutathione peroxidase (GPx) was measured using [12]. This method reduces hydrogen peroxide (H_2_O_2_) formation in reduced glutathione (GSH). The technique consists of incubating in a water bath at 25°C for 5 min, adding 0.2 mL of H_2_O_2_ (1.3 mM) to start the reaction, and after 10 min, adding 1 mL of trichloroacetic acid (TCA) (1%) to stop it. The mixture should include 0.2 mL of the homogenate, 0.4 mL of GSH (0.1 mM), and 0.2 mL of Tris-buffered saline (TBS) buffer solution (50 mM Tris, 150 mM NaCl, pH 7.4). After 30 min on ice, the mixture was centrifuged at 100 × *g* for 10 min; the final mix consisted of 0.48 mL of supernatant, 2.2 mL of TBS booster solution, and 0.32 mL of DTNB (1 mM). At 412 nm, the optical density is red. The CAT activity at 240 nm was calculated by measuring the decrease in hydrogen peroxide levels. The reaction mixture consisted of 1.9 mL of 50 mM phosphate buffer (pH 7.0), 1.0 mL of 5 mM H_2_O_2_, and 100 μL of tissue supernatant.

The absorbance was measured every 15 s for 1 min. The Marklund and Marklund [59] method was based on its ability to inhibit Pyrogallol autoxidation in the presence of EDTA. In a final volume of 1 ml, the assay included 850 μl of Tris HCl buffer (50 mM, pH: 8.2), 20 μL of the sample, 100 μL of EDTA, and 50 μL of Pyrogallol (2.5 mM in 10 mM of HCl). The change in absorbance at 420 nm was measured every minute over 5 min. Malondialdehyde (MDA) was measured to determine the levels of lipid peroxidation (LPO) using the methodology described in Fatima *et al*. [60], Zhao *et al*. [61]. The test mixture contained 1 mL of 0.67% thiobarbituric acid, 1 mL of 5% TCA, and 1 mL of supernatant. The mixture was centrifuged at 100× *g*) for 10 min, heated at 95°C for 40 min, and then cooled with a supernatant absorbance measurement at 532 nm [58].

### Statistical analysis

All statistical analyses were conducted using IBM SPSS Statistics 20.0 (IBM Corp., NY, USA) to ensure accuracy and reliability. The Shapiro–Wilk test was applied to assess whether each variable followed a normal distribution, ensuring the appropriate application of parametric or non-parametric methods. Results were expressed as mean ± standard error (SE), offering a clear depiction of central tendency and variability. A multi-way multivariate analysis of variance (MANOVA) was used to assess differences across groups, such as exposed and non-exposed cattle, for multiple dependent variables simultaneously. This method accounted for potential interdependencies among variables, providing robust group comparisons. Following significant MANOVA results, Tukey’s Honest Significant Difference test was performed to identify specific pairwise differences among group means while minimizing the risk of Type I errors. Pearson’s correlation coefficients were calculated to examine relationships between continuous variables, including biochemical markers (e.g., ALT and AST) and oxidative stress parameters (e.g., GPx, CAT, SOD). This analysis provided insights into potential mechanistic links and interdependencies. Statistical significance was determined at p < 0.05, with highly significant results flagged at p < 0.001. Trends and group differences were visualized using graphs and tables to emphasize key findings and support the statistical conclusions.

## RESULTS

### Biochemical indices

Tables 2 and 3 present the average concentrations of blood biochemical indices related to hepatic and renal functions, as well as lipid and glucose metabolism, for different sampling groups. ALT, AST, ALP, GGT, TP, TC, and LDH significantly differed between groups (p < 0.05, p < 0.001). The non-exposed animal group (NEG) had lower concentrations of ALT, AST, ALP, and LDH than the other groups, with the exposed animal groups (EG 1, EG 2, and EG 3) having the highest levels during the summer months. The non-EG had significantly higher concentrations of TP and TC. The remaining parameters (GLU, ALB, TG, TBIL, and CR) showed no significant variation among the animals. In contrast, cows had higher blood biochemical parameters than male cows (Tables 2 and 3).

**Table 2:** Mean concentrations of blood biochemical indices of exposed and control cattle (n = 40).

Blood biochemical indices	Cattle groups				Total (n = 40)	Significance

	NEG (n = 10)	EG 1 (n = 10)	EG 2 (n = 10)	EG 3 (n = 10)		
ALT (IU/L)	32.10 ± 9.58^a^	44.60 ± 7.24^b^	54.50 ± 10.88^c^	48.50 ± 9.10^b,c^	44.92 ± 12.19	***
AST (IU/L)	102.90 ± 23.46^a^	134.50 ± 24.55^b^	153.90 ± 9.35^b^	137.80 ± 24.15^b^	132.27 ± 27.77	***
ALP (IU/L)	327.20 ± 86.97^b^	530.10 ± 30.26^c^	455.80 ± 92.71^a,c^	398.20 ± 95.33^b,a^	427.82 ± 108.35	***
GGT (IU/L)	15.10 ± 3.69^a^	19.50 ± 4.27^b^	21.60 ± 2.41^b^	20.50 ± 2.36^b^	19.17 ± 4.03^b^	***
TP (g/L)	70.0 ± 3.62^a^	67.80 ± 3.11^a,b^	65.50 ± 2.59^b^	67.70 ± 2.75^a,b^	67.75 ± 3.34	*
GLU (mmol/L)	3.26 ± 0.64	3.05 ± 0.54	2.87 ± 0.47	2.84 ± 0.41	3.00 ± 0.53	ns
ALB (g/L)	32.30 ± 2.71	31.00 ± 2.10	30.10 ± 1.72	30.30 ± 1.88	30.92.2.23	Ns
TC (mmol/L)	2.16 ± 0.22^a^	2.00 ± 0.21^a,b^	1.87 ± 0.18^b^	2.07 ± 0.10^a,b^	2.02 ± 0.21	*
TG (mmol/L)	0.17 ± 0.03	0.15 ± 0.02	0.14 ± 0.02	0.15 ± 0.02	0.15 ± 0.02	Ns
TBIL (μmol/L)	3.26 ± 1.67	3.50 ± 1.31	3.67 ± 1.23	3.67 ± 1.21	3.52 ± 1.32	Ns
CR (μmol/L)	127.00 ± 29.07	126.00 ± 18.97	117.00 ± 21.10	113.00 ± 18.88	120.75 ± 22.34	Ns
LDH (U/L)	2752.20 ± 331.18^a^	3677.70 ± 662.71^b^	4382.50 ± 372.84^c^	4112.20 ± 189.39^b,c^	3731.15 ± 747.99	***

NEG=Non-exposed group, EG1=Cyanotoxins during June, EG2=Cyanotoxins during July, EG2=Cyanotoxins during August. ALT=Alanine aminotransferase, AST=Aspartate transaminase, ALP=Alkaline phosphatase, GGT=Glutamyltransferase, TP=Total protein, GLU=Glucose, ALB=Albumin, TC=Total cholesterol, TG=Triglyceride, TBIL=Total bilirubin, CR=Creatinine, LDH=Lactate dehydrogenase. abc Means in the same row without common letter are different at p < 0.05.

**Table 3:** Mean concentrations of blood biochemical indices in each exposed and control cattle according to the groups and the sex (n = 40).

Blood biochemical indices	Cattle groups								Total (n = 40)	

	NEG		EG 1		EG 2		EG 3			
				
	F (n = 5)	M (n = 5)	F (n = 5)	M (n = 5)	F (n = 5)	M (n = 5)	F (n = 5)	M (n = 5)	F (n = 20)	M (n = 2)
ALT (IU/L)	37.6 ± 6.91	26.6 ± 9.12	49 ± 6.67	40.2 ± 5	63 ± 8.36	46 ± 4	55.8 ± 6.61	41.2 ± 3.11	51.35 ± 11.64	38.5 ± 9.11
AST (IU/L)	111 ± 24.96	94.8 ± 21.25	143.6 ± 7.66	125.4 ± 33	160 ± 8.71	147.8 ± 5.26	155.4 ± 6.18	120.2 ± 22.35	142.5 ± 23.52	122 ± 28.45
ALP (IU/L)	348 ± 101.85	306.4 ± 74.58	546.4 ± 35.74	513.8 ± 10.91	480.4 ± 73.3	431.2 ± 11.59	423.4 ± 87.61	373 ± 105.75	449.55 ± 103.98	406.6 ± 110.88
GGT (IU/L)	17.2 ± 2.86	13 ± 3.39	21.8 ± 1.92	17.2 ± 4.91	22.4 ± 2.40	20.8 ± 2.38	21.6 ± 2.07	19.40 ± 2.3	20.75 ± 3.02	17.60 ± 4.35
TP (g/L)	68.60 ± 3.91	71.4 ± 3.04	67.0 ± 3.24	68.6 ± 3.13	64.80 ± 1.92	66.2 ± 3.19	68.2 ± 2.94	67.2 ± 2.77	67.15 ± 3.21	68.35 ± 3.43
GLU (mmol/L)	3.28 ± 0.78	3.24 ± 0.55	2.82 ± 0.47	3.28 ± 0.54	2.68 ± 0.4	3.06 ± 0.5	2.70 ± 0.24	2.89 ± 0.52	2.87 ± 0.53	3.14 ± 0.50
ALB (g/L)	32.4 ± 2.88	32.20 ± 2.86	31.40 ± 2.60	30.60 ± 1.67	30.40 ± 1.81	29.80 ± 1.78	31 ± 2.34	29.6 ± 1.14	31.3 ± 2.36	30.55 ± 20.8
TC (mmol/L)	2.14 ± 0.2	2.18 ± 0.25	1.96 ± 0.24	2.04 ± 0.20	1.84 ± 0.24	1.90 ± 0.14	2.04 ± 0.11	2.10 ± 00.10	1.99 ± 0.22	2.05 ± 0.20
TG (mmol/L)	0.17 ± 0.05	0.17 ± 0.01	0.15 ± 0.02	0.16 ± 0.01	0.12 ± 0.03	0.15 ± 0.01	0.15 ± 0.02	0.15 ± 0.01	0.15 ± 0.03	0.16 ± 0.01
TBIL (μmol/L)	3.88 ± 1.84	2.64 ± 1.4	3.90 ± 1.5	3.10 ± 1.11	4.08 ± 1.34	3.26 ± 1.07	4.12 ± 1.31	3.22 ± 1.04	3.99 ± 1.39	3.05 ± 1.10
CR (μmol/L)	136 ± 27.01	118 ± 31.14	132 ± 14.83	120 ± 22.36	124 ± 15.16	110 ± 25.49	118 ± 16.43	108 ± 21.67	127.5 ± 18.88	114 ± 23.92
LDH (U/L)	2853.8 ± 215.31	2650.6 ± 417.87	3976.6 ± 716.38	3378.8 ± 501.61	4580 ± 342.80	4185 ± 312.66	4146 ± 136.76	4078.4 ± 243.2	3889.1 ± 757.32	3573.2 ± 722.75

NEG=Non-exposed group, EG1=Cyanotoxins during June, EG2=Cyanotoxins during July, EG2=Exposed group to cytotoxins during August month, ALT=Alanine aminotransferase, AST=Aspartate transaminase, ALP=Alkaline phosphatase, GGT=Glutamyltransferase, TP=Total protein, GLU=Glucose, ALB=Albumin, TC=Total cholesterol, TG=Triglyceride, TBIL=Total bilirubin, CR=Creatinine, LDH=Lactate dehydrogenase

### Histopathology evaluation

#### Macroscopic assessment

**Figure 2:**
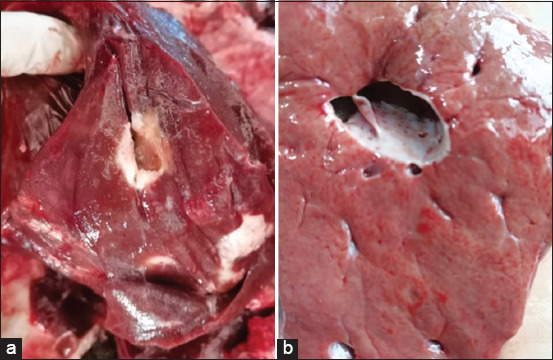
Normal-looking liver parenchyma. (a) Duct bile sclerosis and (b) dilated bile ducts.

Visual inspection, palpation, and incision of the bile ducts in the NEG and EG1 revealed that they were homogeneous and ordinary in appearance. The livers of EG2 cattle showed biliary duct dilatation, which was sometimes discrete, with calcified hepatic duct walls and normal parenchyma. Biliary dilation was associated with biliary wall fibrosis in EG3 (Figure 2).

#### Microscopic findings

**Figure 3:**
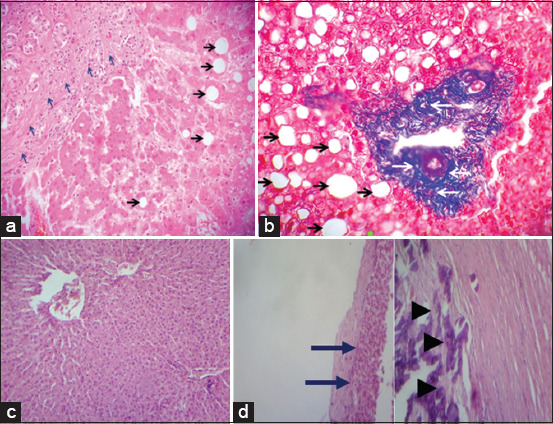
Liver histo-pathology of cattle exposed to microcystins in Lake des Oiseaux. (a) Black arrows: Macrovacuolar steatosis. Blue arrows: Portal fibrosis and mononuclear inflammatory infiltrate (Color hematoxylin and eosin [H&E]) (100×). (b) Black arrows: Macrovacuolar steatosis. White arrows: Portal fibrosis. (MASSON Trichrome histochemistry) (100×). (c) Unremarkable hepatic parenchyma (Color H&E) (100×). (d) Blue arrows: Biliary dilation–Black arrows: fibrosis and calcification (Color H&E) (100×).

The hepatocytes of the control group (NEG) had a normal shape, with dense cytoplasm and centrally located nuclei. There were no significant histological changes detected in EG 1 (Figure 3), whereas in EGs EG 2 and EG 3, liver changes were observed to varying degrees in comparison to the control group (NEG) (Tables 4 and 5).

**Table 4:** Means of inflammation, fibrosis, and steatosis indexes in cattle according to the groups (exposition period).

Lesion index	Cattle groups				Total (n = 40)	Significance

	NEG (n = 10)	EG 1 (n = 10)	EG 2 (n = 10)	EG3 (n = 10)		
Inflammation grade	0.1 ± 0.31^a^	0.40 ± 0.51^a,b^	1.0 ± 0.66^b^	0.8 ± 0.78^b^	0.57 ± 0.67	**
Fibrosis grade	0.1 ± 0.31^a^	0.40 ± 0.51^a,b^	1.0 ± 0.66^b^	0.8 ± 0.78^b^	0.57 ± 0.67	**
Steatosis grade	00^a^	0.40 ± 0.51^a,b^	1.7 ± 1.05^c^	1.0 ± 0.66^b,c^	0.77 ± 0.91	***

^abc^Means in the same row without common letter are different at p < 0.05.

*The lesions were assessed using an objective index on a numerical scale of 0 – 4: 0=No lesion, 1=Slightly diffuse lesion, 2=Intermediate diffuse lesion, 3=Diffuse lesion, and 4=Very diffuse lesion (*p < 0.05,

** p < 0.01).

**Table 5:** Liver lesion levels in the cattle according to the groups and the sex.

Lesion index	Cattle groups								Total	

	NEG		EG 1		EG 2		EG3			
			
	F (n = 5)	M (n = 5)	F (n = 5)	M (n = 5)	F (n = 5)	M (n = 5)	F (n = 5)	M (n = 5)	F (n = 20)	M (n = 20)
Inflammation grade*♣	2 ± 0.44	0	2 ± 0.44	0.6 ± 0.54	1.4 ± 0.54	0.6 ± 0.54	1.2 ± 0.83	0.4 ± 0.54	0.75 ± 0.78	0.40 ± 0.50
Fibrosis grade*♣	0.2 ± 0.44	0	0.2 ± 0.4	0.6 ± 0.54	1.4 ± 0.54	0.6 ± 0.5	1.2 ± 0.83	0.4 ± 0.54	0.75 ± 0.78	0.40 ± 0.50
Steatosis grade *♣	0	0	0.6 ± 0.54	0.2 ± 0.44	2.2 ± 0.83	1.2 ± 1.09	1.4 ± 0.54	0.6 ± 0.54	1.05 ± 0.99	0.50 ± 0.76

♣The objective index with a numerical scale of 0–4 was used to assess the lesions as follows: 0=No lesion, 1=Slightly diffuse lesion, 2=Intermediate diffuse lesion, 3=Diffuse lesion, and 4=Very diffuse lesion.

* Significance of group × sex interaction (inflammation grade=0.05, fibrosis grade=0.05, steatosis grade=0.492).

### Biochemical oxidative stress

#### Antioxidant enzymes and GSH

**Figure 4:**
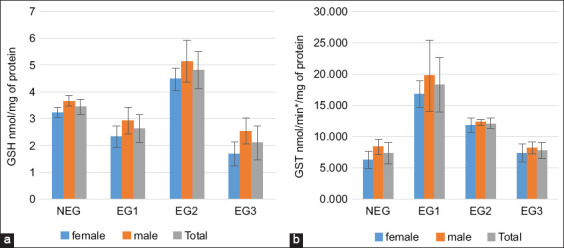
(a) Glutathione and (b) glutathione S-transferase levels in cattle livers exposed to microcystins in Lake des Oiseaux. The values are expressed as mean ± standard error. The number of measurements performed in each group was three (n = 10/group).

Cattle livers exposed to lake water containing the cyanobacterial toxin “microcystins” showed significant fluctuations in GSH levels (p < 0.001; Figure 4). The second group had the highest value (4.80 ± 0.68 nmol/mg of protein), whereas the livers of the first and third EGs showed a reduction. The liver showed significant induction of the metabolizing enzyme GST (p < 0.001; Figure 4). The first EG’s cattle liver had the highest increase (18.27 ± 4.34 nmol/min/mg of protein).

**Figure 5:**
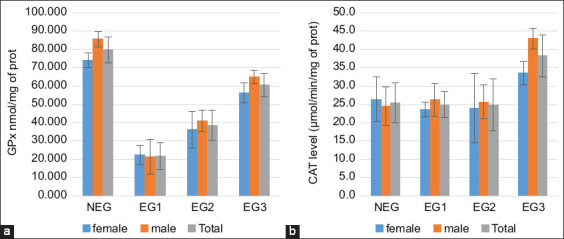
(a) Glutathione peroxidase and (b) catalase in cattle livers exposed to microcystins in Lake des Oiseaux. The values are expressed as mean ± standard error. The number of measurements performed in each group was three (n = 10/group).

Cattle exposed to cyanotoxin-contaminated lake water exhibited a significant variation in GPx levels (p < 0.001; Figure 5). The liver showed a significant decrease in GPx (p < 001) compared with the control group, with the lowest values of 21.87 ± 7.22 nmol GSH/mg protein detected in the first EG. CAT levels in the liver showed significant differences (p < 0.001). Catalase levels were stable in both EG1 and EG2 but increased in the last batches to 38.30 ± 5.72 μmol/min/mg protein (p < 0.001).

#### LPO

**Figure 6:**
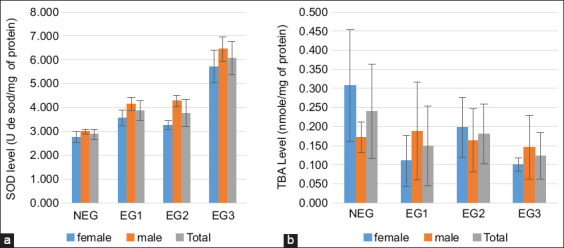
(a) Superoxide dismutase and (b) lipid peroxidation values in cattle livers exposed to microcystins in Lake des Oiseaux. The values are expressed as mean ± standard error. The number of measurements performed in each group was three (n = 10/group).

Figure 6 shows the variation in SOD levels, with an increase observed in all groups of cattle livers. The third EG showed strong activation of the defense enzyme (6.07 ± 0.69 U of sod/mg of protein). Figure 6f shows that LPO levels decreased in all liver groups. The third group (EG3) showed significant variation in LPOs (p < 0.05), with the lowest value of 0.12 ± 0.06 nmol/mg protein (Table 6).

**Table 6:** Mean levels of biochemical oxidative stress indices in cattle according to the groups (exposition period).

biochemical oxidative stress indices	Cattle groups				Total (n = 40)	Significance

	NEG (n = 10)	EG 1 (n = 10)	EG 2 (n = 10)	EG3 (n = 10)		
Glutathione	3.44 ± 0.28^a^	2.63 ± 0.52^b^	4.80 ± 0.68^c^	2.10 ± 0.63^a^	3.24 ± 1.16	***
Glutathione -s- transferase	7.30 ± 1.67^a^	18.27 ± 4.34^b^	12.04 ± 0.85^c^	7.76 ± 1.24^a^	11.34 ± 5.04	***
Glutathione peroxidase	79.87 ± 7.17^a^	21.87 ± 7.22^b^	38.66 ± 8.13^c^	60.56 ± 6.35^d^	50.24 ± 23.27	***
Catalase	25.46 ± 5.51^a^	24.94 ± 3.52^a^	24.85 ± 7.06^a^	38.30 ± 5.72^b^	28.39 ± 7.90	***
Superoxide- dismutase	2.87 ± 0.21^a^	3.85 ± 0.42^b^	3.76 ± 0.56^b^	6.07 ± 0.69^c^	4.14 ± 1.28	***
lipid Peroxidation	0.24 ± 0.12^b^	0.14 ± 0.10^a^,^b^	0.18 ± 0.07^a^,^b^	0.12 ± 0.06^a^	0.17 ± 0.10	*

* The lesions were assessed using an objective index on a numerical scale of 0–4: 0=No lesion, 1=Slightly diffuse lesion, 2=Intermediate diffuse lesion, 3=Diffuse lesion, and 4=Very diffuse lesion (*p < 0.05, **p < 0.01). ^abc^Means in the same row without common letter are different at p < 0.05.

## DISCUSSION

Cyanotoxin ingestion is the most well-known mechanism, whereas inhalation mechanisms are still poorly understood [62], with no conclusive evidence of negative effects [63]. *In vitro* studies on respiratory epithelial cells exposed to CYN115 [64, 65] revealed that the importance of cellular damage increases with exposure time [20, 66]. The cyanobacterial bloom in the study area (Lake des Oiseaux, Algeria) contained six morphotypes of Microcystis with varying individual cell sizes, colony morphology and size, and mucilage characteristics. The total MC content was estimated to be 0.062 mg MC-LR equivalents/g dried bloom, with MC-RR dominating among the 21 MCs [67]. The transfer of MC through the food chain is well understood [68]. The collected data show a high prevalence of MCs in aquatic edible species around the world, confirming that the accumulation of these toxins is a global concern [69, 70]. However, in the past decade, only a few studies have reported MC-RR congener concentrations [71]. According to Funari and Testai [72], acute toxicity is 10 times lower in MC-RR than in MC-LR. However, the presence of two arginine makes this congener more hydrophilic (polar) than other variants, which is important for its toxicokinetic properties [71]. When MC-RR is absorbed, it quickly enters tissues [73, 74] and is metabolized with GSH in the presence of GST [73, 75], which aids in its elimination [21].

Living water from natural eutrophic sources may expose livestock to toxic cyanobacteria. Brena *et al*. [76] examined livestock drinking water sources from ranches in a South American eutrophic subtropical basin where dense blooms of *Microcystis* spp. were found. MC concentrations in cattle drinking water can reach up to 3700 μg/L, indicating a high risk of poisoning. The exposed animals had no significant changes in liver enzymes, but M.C.s serum concentrations increased to 0.11 μg/L. The transmission of cyanotoxins to grazing animals is not limited to water; plants and grains grown near contaminated lakes absorb these toxins and rich animal organisms through direct consumption [77, 78]. A herd of 53 cows and one herd of bulls gained access to stagnant water dominated by toxic Mc from a small river (Ribeira de Oeiras-Portugal). In <19 h, 20 cows died with obvious clinical symptoms; the remaining 29 cows were healthy, and five sick cows died. Clinical and pathological examinations revealed hepatic and renal necrosis [79].

In 2016, wildlife mortality near two dams in the southern and southern central regions of Kruger National Park in South Africa was caused by intense cyanobacterial blooms of *Microcystis* spp. in the dam water [80]. The macroscopic and histopathological findings supported the diagnosis of cyanobacterial poisoning [80, 81]. The Bengis team’s investigation revealed zebra liver severe parenchymal alterations. Cyanotoxin organ toxicity can damage tissues and cells in all animal and human organisms, including glands, gastrointestinal tracts, central nervous system tissues, gonads, blood and vessels, muscles and bones, and embryos [82, 83].

Over the past two decades, more than 100 cattle deaths have been reported at 11 alpine sites in south-east Switzerland. Pathological and histological examinations of the organs revealed acute hepatotoxicosis. The algal community in these waters was dominated by benthic cyanobacteria, specifically *Oscillatoria limosa* and *Phormidium konstantinos* [80, 84]. This study’s findings were similar to those of Manubolu *et al*. [4], who investigated cyanotoxin levels in water samples collected from four different locations in the Baltic Sea over three seasons, including the summer of 2011. The data show the adsorption of toxins from water into the circulation of grazing cattle, with no measurable liver damage caused by cyanotoxin poisoning. Manubolu *et al*. [4] proposed that the rumen microbial flora can protect ruminants (cattle) from cytotoxins. Cattle exposed to low concentrations of MCs degrade toxins: MC-RR 36%, NOD 35%, MC-RR 25%, and MC-LR 8.9%, within 3 h [85]. The results are in agreement with those of other previous studies by [4, 84, 85].

Su *et al*. [85] demonstrated that chronic exposure (14 days) to MC causes prolonged weight loss, per rectum bleeding, extensive colonic ulcers, and shorter colon length, exacerbating the severity of pre-existing colitis [86, 87]. In 2014, Svirčev *et al*. [88] published a review on the relationship between the incidence of 13 cancers in Serbia over 10 years and the presence of cyanobacterial blooms in reservoirs that supply drinking water. There was a significantly higher incidence of 11 malignant cancers: brain, heart, mediastinum, pleura, ovary, testis, stomach, liver, colo-rectum, retroperitoneum, and peritoneum. Previous studies reported by Brena *et al*. [76] and Lee *et al*. [89] showed a significant link between freshwater cyanobacterial blooms and an increased risk of death from non-alcoholic liver disease. These organizations identified cyanobacterial blooms as a major environmental and public health concern affecting aquafarms, major drinking water supplies, freshwater bodies, and agriculture worldwide.

This study confirms previous findings by Zaidi *et al*. [58] and Lone *et al*. [90] that MC exposure inhibits the activities of endogenous antioxidant enzymes such as GPx and CAT. MC-LR can suppress GST expression and activity [91]. A significant variation in GPx was detected in the liver of cattle. GPx activity is also affected by dietary selenium intake and reduced glutathione (GSH) levels, which are involved in the GST-catalyzed reaction. Because GPx relies on GSH as a reduction factor for hydroperoxide detoxification, its value may decrease if GSH is unavailable [14]. In 2023, Zhang *et al*. [92] collected data from 67 studies and discovered that the activities of MDA and lactoperoxidase (LPO) always increased; under all conditions of MC-LR exposure, growth may be regarded as a highly sensitive indicator of MC-LR toxicity in mammals and fish. Cyanobacteria toxicity can activate apoptosis enzymatic activity and increase proinflammatory mediator levels [30], resulting in oxidative stress, apoptosis, inflammation, and the synthesis of harmful secondary metabolites [82, 93–95]. Furthermore, exposure to cytotoxins causes serious DNA changes [78]. Finally, MC causes brain damage and functional impairment in *Lithobates catesbeianus* tadpoles by increasing oxidative stress and inflammation levels [92, 96].

This study had a secondary preventive goal of considering the findings and the health conditions in and around Lake des Oiseaux. The lake’s MC concentrations may soon exceed the WHO guidelines of 1 μg/L for drinking water, 4.2 μg/L for cattle, and 3.9 μg/L for sheep due to uncontrolled growth [96, 97]. Crucial points and solutions must be prioritized to avoid such fears and significantly reduce the prevalence of any negative impact on humans. Protected wells that draw water from deep aquifers can provide MC-free water. The surface water intake settling and filtration systems also remove toxic cyanobacterial cells. Monitoring MC levels and preventing cattle access should be implemented when toxins exceed safe limits. Furthermore, raising farmers’ awareness of the risks associated with cyanobacterial blooms can help prevent cattle poisoning [98].

## CONCLUSION

This study provides critical insights into the long-term effects of microcystin exposure on cattle health, emphasizing the interconnected risks to animals, humans, and the environment under the “One Health” framework. The findings demonstrate significant biochemical, histopathological, and oxidative stress alterations in exposed cattle. Elevated liver enzyme levels (ALT, AST, ALP and GGT), oxidative stress markers (LPO), and reduced antioxidant defenses (GPx, CAT and GSH) collectively highlight the toxic impact of microcystins on hepatic function and redox balance. Histological changes, including macrovacuolar steatosis, fibrosis, and bile duct dilatation, further corroborate the adverse effects of chronic cyanotoxin exposure.

The study’s strength lies in its real-life ecological setting and comprehensive analysis, integrating biochemical, histological, and oxidative stress assessments. This multi-faceted approach offers a holistic understanding of microcystin toxicity and establishes a robust baseline for further investigations. Moreover, the use of cattle as sentinel organisms reinforces the study’s relevance to environmental and public health concerns, particularly in regions dependent on livestock for sustenance.

However, the study has certain limitations. The sample size, while sufficient for initial analysis, may not fully capture the variability across broader populations or diverse ecological conditions. In addition, the research focuses solely on biochemical and histological parameters, limiting insights into potential systemic or reproductive effects of microcystin exposure. The absence of longitudinal monitoring beyond a single summer season also restricts understanding of chronic and cumulative impacts.

Future research should expand the scope to include larger and more diverse populations, long-term monitoring, and the exploration of systemic effects, including impacts on reproduction and offspring health. Investigating effective mitigation strategies, such as water treatment technologies and the development of biosensors for real-time cyanotoxin detection, will be crucial. Expanding studies to evaluate the potential transmission of cyanotoxins through the food chain can also enhance understanding of risks to human health.

These findings underscore the urgency of implementing preventive measures, including improved water quality management and farmer education, to safeguard both livestock and human populations.

## DATA AVAILABILITY

The supplementary data of this study can be made available by the corresponding author on request.

## AUTHORS’ CONTRIBUTIONS

MB, HN, OL, and ZW: Conceived, designed, and coordinated the study. MB, FS, and ZH: Principal investigators. MB and AA: Designed data collection tools. HN and OL: Supervised field sampling, data collection, laboratory work, and data entry. MB, AA, ZH, HN, and ZW: Statistical analysis, interpretation of results, and drafted the manuscript. All authors have read and approved the final version of the manuscript.
